# Carotenoids, Phenolic Compounds and Tocopherols Contribute to the Antioxidative Properties of Some Microalgae Species Grown on Industrial Wastewater

**DOI:** 10.3390/md13127069

**Published:** 2015-12-11

**Authors:** Hamed Safafar, Jonathan van Wagenen, Per Møller, Charlotte Jacobsen

**Affiliations:** 1 National Food Institute (DTU Food), Technical University of Denmark, Søltofts Plads, Building 221, 2800 Kongens Lyngby, Denmark; chja@food.dtu.dk; 2 Residual Resources Engineering (RRE), DTU Environment, Technical University of Denmark, Miljøvej, Building 113, 2800 Kongens Lyngby, Denmark; jovw@env.dtu.dk; 3 Kalundborg Municipality, Udviklingsstaben Hareskovvej 14-16, 4400 Kalundborg, Denmark; per.moller@kalundborg.dk

**Keywords:** microalgae, phenolic compounds, antioxidants, wastewater, carotenoids, tocopherols, DPPH, FRAP

## Abstract

This study aimed at investigating the potential of microalgae species grown on industrial waste water as a new source of natural antioxidants. Six microalgae from different classes, including *Phaeodactylum* sp. (Bacillariophyceae), *Nannochloropsis* sp. (Eustigmatophyceae), *Chlorella* sp., *Dunaniella* sp., and *Desmodesmus* sp. (Chlorophyta), were screened for their antioxidant properties using different *in vitro* assays. Natural antioxidants, including pigments, phenolics, and tocopherols, were measured in methanolic extracts of microalgae biomass. Highest and lowest concentrations of pigments, phenolic compounds, and tocopherols were found in *Desmodesmus* sp. and *Phaeodactylum tricornuotom* microalgae species, respectively. The results of each assay were correlated to the content of natural antioxidants in microalgae biomass. Phenolic compounds were found as major contributors to the antioxidant activity in all antioxidant tests while carotenoids were found to contribute to the 1,1-diphenyl-2-picryl-hydrazil (DPPH) radical scavenging activity, ferrous reduction power (FRAP), and ABTS-radical scavenging capacity activity. *Desmodesmus* sp. biomass represented a potentially rich source of natural antioxidants, such as carotenoids (lutein), tocopherols, and phenolic compounds when cultivated on industrial waste water as the main nutrient source.

## 1. Introduction

Algae are one of the oldest living organisms of planet earth. Microalgae can grow in quite different environments, like sea, and desert [1]. In recent years algae have been in the center of interest as a sustainable, rich source of bioactive compounds, like phenolic compounds, fatty acids, amino acids, and carotenoids. There has also been a global trend to replace artificial antioxidants with natural antioxidants during the past two decades. Antioxidants are increasingly being used in food supplements as bioactive compounds and in functional foods to increase their shelf life and prevent unwanted lipid oxidation. Nearly all commercially available natural antioxidants are derived from terrestrial plants [2]. It is, however, believed that microalgae could be an alternative resource of natural antioxidants as they are much more diverse than other sources like plants [3]. The global market for micro-algae-based food and feed supplements/nutraceuticals is well developed and with a great potential for growth, so investigation of antioxidative properties and natural antioxidant composition of microalgae biomass is important. There are a number of reports on the evaluation of antioxidant activity of some microalgae and cyanobacteria species belonging to the genera of *Botryococcus* [4], *Chlorella* [5,6,7], *Dunaliella* [8], *Nostoc* [9], *Phaeodactylum* [10], *Spirulina* [11,12], *Nannochloropsis, Chaetoceros* [13], *Halochlorococcum*, *Oltamannsiellopsis* [14], and *Navicula clavata* [7].

Carotenoids are a family of yellow to orange-red terpenoid pigments synthesized by photosynthetic organisms as well as some bacteria and fungi [15]. Carotenoids can act as antioxidants by scavenging and deactivating free radicals [16]. Carotenoids include two classes; xanthophylls, which contain oxygen, and carotenes, which are purely hydrocarbons and contain no oxygen. All xanthophylls synthesized by higher plants e.g., violaxanthin, antheraxanthin, zeaxanthin, neoxanthin, and lutein, can also be synthesized by green microalgae; however, these possess additional xanthophylls, e.g., loroxanthin, astaxanthin, and canthaxanthin. Diatoxanthin, diadinoxanthin, and fucoxanthin can also be produced by brown algae or diatoms [15]. Several studies have shown that carotenoids contribute significantly to the total antioxidant capacity of microalgae [16,17,18].

The term “polyphenol” includes more than 8000 compounds with great diversity in structure. They can be divided into 10 different classes depending on their basic chemical structure [19]. Phenolic compounds are recognized as important natural antioxidants. Polyphenols act as antioxidant through single electron transfer and through hydrogen atom transfer [16]. Some studies suggest that the content of phenolic substances in microalgae is lower than or equal to the minimum amounts reported for terrestrial plants [4], and just include phenolic acids. However some recent studies showed that several classes of flavonoids, such as isoflavones, flavanones, flavonols, and dihydrochalcones can also be found in microalgae [18]. This clearly demonstrates that microalgae are able to produce also more complex phenolic compounds, so characterization and identification of phenolic compounds in microalgae are required, especially as they may contain novel phenolic compounds [19].

There are only few published studies regarding the identification and quantification of phenolic composition in microalgae species [8,11,20,21]. Abd El-Baky *et al*. [20] found phenolic compounds including gallate, chlorogenate, cinnamate, pinostrobate, and *p*-OH-benzoates in *Spirulina* sp. Other researchers reported salicylic, *trans*-cinnamic, synapic, chlorogenic, and caffeic acids as the main phenolic acids in this microalgae species [12]. The results of these studies show the intensive effects of growth media on the phenolic composition of microalgae species. In a very recent UPLC-MS/MS study, simple phenolics and hydroxycinnamic acids (ferulic acid and *p*-coumaric acid) were detected in *Chlorella vulgaris*, *Haematococcus pluvialis*, *Diacronema lutheri*, *Phaeodactylum tricornutum*, *Tetraselmis suecica*, and *Porphyridium purpureum* microalgae species [22].

There are many studies concerning the screening of microalgae species based on their antioxidative properties by using different *in vivo* and *in vitro* assays. Goiris *et al.* [16] screened 32 microalgal biomass samples for their antioxidant capacity using three antioxidant assays, and both total phenolic content and carotenoid content were measured. The study revealed that industrially-cultivated samples of *Tetraselmis suecica*, *Botryococcus braunii*, *Neochloris oleoabundans*, *Isochrysis* sp., *Chlorella vulgaris*, and *Phaeodactylum tricornutum* possessed the highest antioxidant capacities and, thus, could be potential new sources of natural antioxidants. The results also showed that both phenolic and carotenoids contributed significantly to the antioxidant capacity of microalgae.

The main pollutants in different wastewater sources are nitrogen (N) and phosphorus (P) in different forms, which on the other hand, are necessary nutrients for algae growth. Recent studies have shown that some microalgae can grow on wastewater, uptake the nutrients such as N and P, reduce the biological oxygen demand (BOD) and produce biomass, which can be used for different purposes [23]. Wastewater can provide water medium as well as nearly all necessary nutrients for cultivation of microalgae. Combination of wastewater treatment and algae cultivation could be a feasible, environmentally-friendly approach for sustainable production of algae-based bioactive compounds [23]. The bio-refinery approach consists of sustainable production of biomass through an integrated process. As an example, use of these strategies may offer an inexpensive alternative to the conventional technological routes of production of natural pigments [24].

The aim of this study was to investigate natural antioxidant composition and antioxidative properties of some microalgae species from different classes including *Phaeodactylum* sp. (Bacillariophyceae), *Nannochloropsis* sp. (Eustigmatophyceae), *Chlorella* sp., *Dunaniella*, and *Desmodesmus* (Chlorophyta) which were grown autotrophically on industrial waste water.

## 2. Results and Discussion

### 2.1. Extraction of Phenolics and Carotenoids

**Figure 1:**
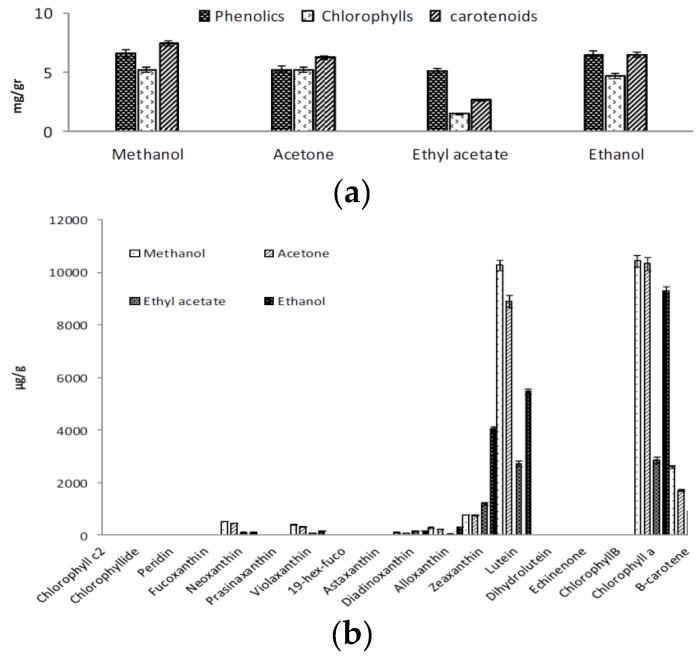
(**a**) Evaluation of extraction yield of different solvents for phenolic compounds and pigments; and (**b**) evaluation of effects of different solvents on carotenoids content and composition.

In microalgae, carotenoids and phenolic compounds are surrounded by cell wall and, therefore, procedures that can break down cell walls with minimum risk of damage are needed. An efficient extraction requires that the solvent penetrates into the cell and dissolves the target compounds corresponding to the polarity. Many different non-conventional extraction methods including electrical pulsed electric fields (PEF), high-voltage electrical discharges (HVED), high-pressure homogenization, ultrasounds, microwaves, sub- and supercritical fluid extraction, have been proposed as suitable techniques to achieve this purpose [25]. Combination of these techniques with the solvent extraction increase the yield of extraction. In a recent study, a high level of extraction of pigments and other bioactive compounds is reported by using the combination of pulsed electric field assisted extraction and solvent extraction in biomass of *Nannochloropsis* spp. [26]. Ultrasound-assisted solvent extraction is reported as a promising tool to recover high-added value compounds from the microalgae *Nannochloropsis* spp. [27]. Low temperature sonication could enhance the cell rupture efficiency, without negative mechanical or heat induced effects on sensitive carotenoids. In this study, we evaluated the combination of sonication technique with four common extraction solvents for the extraction of both carotenoids and phenolics in samples of *Chlorella sorokiniana* by procedures described at Section 3.1 and Section 3.2 of this paper. In the ultrasonic process, the microalgal cells are disrupted by shock waves from cavitation bubbles, enhancing the liberation of valuable compounds. The sonication process utilizes the cavitation to disrupt the cell wall and results in the physical effects of micro-turbulence and velocity/pressure shockwaves. Micro-turbulence provides intense mixing, while shockwaves cause disruption of the cell wall [25]. Methanol extraction showed the highest concentration of both carotenoids and phenolics (Figure 1a). For all of the pigments higher levels of extraction were achieved by using methanol (Figure 1b), even for beta carotene, which as a hydrocarbon lacking functional groups, is very lipophilic and more soluble in acetone [28].

Methanol is one the most favored solvents which is used for the extraction of polar compounds such as phenolic compounds and flavonoids. It has previously been shown that methanol extract of microalgae has more antioxidative power compared to extracts obtained with other common solvents [6,7,29] and it was also claimed that methanol can disintegrate cell membranes more than other solvents [30]. In the present study pigments were extracted from dried samples, which prevent the risk of degradation of pigments. Thus, development of chlorophyll derivatives (e.g., pheopigment) arising normally from sample processing was reduced or prevented.

### 2.2. Total Phenolics, Flavonoids, and Phenolic Composition

The phenolic contents based on Folin method varied from 7.72 ± 0.08 to 3.16 ± 0.04 (mg/g GAE) with statistically significant differences among the species (Table 1). The highest and lowest concentrations were found in *Desmodesmus* (De.S) and P.T respectively. There were no significant differences between C.S1 and C.S2, so light intensity did not show significant effect on the phenolic content in *Chlorella sorokinia* species (C.S1 and C.S2). Comparison of the results by simple regression shows a statistically significant relationship between phenolic composition by HPLC and total phenolic compounds at the 95.0% confidence level. The correlation coefficient equals 0.88 which indicates a strong relationship between the results, indicating that identified phenolic acids are the main contributors to the compounds measured by the Folin method. Our result falls within the range given by previous reports [3,7,9,12,27,31,32,33,34,35]. Hajimahmoodi *et al.* [33] reported that *Chorella vulgaris* had the highest total phenolic content among samples of water extracts from 12 strains of microalgae. The study also showed that phenolic compounds were major contributors to the microalgae antioxidant capacity. Production of phenolics as well as other antioxidant compounds in microalgae depends on the growth conditions and stresses such as oxidative stress, so it shall be considered when the results are being compared to other studies.

**Table 1:** Total phenolics, flavonoids, carotenoids, and tocopherols in microalgae biomass.

Species	Total Phenolics (mg/g) *	Total Tocopherols (µg/g)	Total Carotenoids (mg/g)	Total Flavonoids (mg/g) **
De.S	7.72 ± 0.08 a	361.9 ± 23 a	6.70 ± 0.01 a	4.03 ± 1.10 a
Du.S	4.52 ± 0.05 d	125.2 ± 23.5 b	4.83 ± 0.01 d	3.61 ± 1.09 a
N.L	5.78 ± 0.12 c	21.18 ± 0.05 d,e	2.56 ± 0.02 g,f	2.6 ± 0.30 a,b
P.T	3.16 ± 0.04 f	13.12 ± 0.01 e	4.60 ± 0.03 e	0.84 ± 0.12 a
N.S	6.45 ± 0.25 b	44.08 ± 3.11 c	5.296 ± 0.01 b	3.18 ± 0.59 a
C.S1	5.86 ± 0.06 c	34.13 ± 0.37 c,d	4.978 ± 0.06 c	2.49 ± 0.7 a,b
C.S2	5.76 ± 0.12 c	33.74 ± 0.27 c,d,e	2.92 ± 0.15 f	2.41 ± 0.9 a,b

Values are given as mean (*n* = 3) ± standard deviation (absolute value). For each column, same letters indicate similar values (*p* < 0.05); * As gallic acid equivalent; ** As quercetine equivalent.

Our results for identified phenolic (HPLC) varied from 10.07 ± 0.04 µg/g for N.S to 5.10 ± 0.12 µg/g for P.T. as shown in Table 2. Only simple phenolic acids were identified by using reference standards, so the results did not include more complex phenolics. Identified phenolic acids include gallic acid, 2,5-dihydroxy benzoic acid, 3,4-dihydroxy benzoic acid, caffeic acid, ferulic acid, *p*-coumaric acid, salycilic acid, and cinnamic acid (Table 2). In this study we detected hydroxy cinnamic acids in green algae including De.S, Du.S, C.S1, and C.S2, which belong to the same class. All samples excluding P.T contain ferulic acid while 3, 4 dihydroxy benzoic acid was only found in PT and N.S., and *p-*coumaric acid was identified in all samples excluding N.L. Two batches of *Chlorella* *sorokiniana* grown in different light intensities had the same phenolic acid profile, but the total identified phenolic acids was slightly higher in the sample grown in normal light intensity (C.S2).Characterization of phenolic acids in microalgae species has been carried out in other studies, which can confirm some of the results of this study. One study reported the presence of highly-polar phenolic compounds of C6-C11 or C6 phenolic skeletons [11], single phenols including protocatechuic, *p*-hydroxybenzoic, vanillic, syringic, caffeic, chlorogenic acid, 4-hydroxybenzaldehyde, and 4-dihydroxybenzaldehyde in *Spirulina* [20], hydroxycinnamic acids (ferulic acid, *p*-coumaric acid) in *Chlorella vulgaris*, *Haematococcus* *pluvialis*, *Diacronema lutheri, Phaeodactylum* sp.*, Tetraselmis suecica*, and *Porphyridium purpureum*, and *p*-hydroxybenzoic, protocatechuic, vanillic, syringic, caffeic, and chlorogenic acid; 4-hydroxybenzaldehyde and 3,4-dihydroxybenzaldehyde in *Spongiochloris spongiosa* and *Spirulina platensis, Anabaena doliolum, Nostoc* sp.*,* and *Cylindrospermum* sp. [22].

**Table 2:** Identified phenolic composition of microalgae biomass.

Phenolic Compounds (µg/g)	De.S	Du.S	N.L	P.T	N.S	C.S1	C.S2
Gallic acid	4.32 ± 0.01	nd	2.30 ± 0.02	nd	2.75 ± 0.03	nd	nd
2,5 dihydroxy benzoic acid	nd	nd	nd	nd	nd	nd	nd
3,4 dihydroxy benzoic acid	nd	nd	nd	1.64 ± 0.02	2.90 ± 0.07	nd	nd
Caffeic acid	1.11 ± 0.01	1.34 ± 0.04	1.37 ± 0.04	nd	nd	3.81 ± 0.03	3.12 ± 0.20
Ferulic acid	1.41 ± 0.04	4.07 ± 0.03	2.45 ± 0.04	nd	2.90 ± 0.05	2.81 ± 0.03	2.80 ± 0.20
*p*-Coumaric acid	1.91 ± 0.01	0.67 ± 0.02	nd	1.56 ± 0.12	0.29 ± 0.09	1.97 ± 0.05	1.16 ± 0.09
Salycilic acid	nd	nd	0.55 ± 0.07	1.91 ± 0.20	1.32 ± 0.01	nd	nd
Cinnamic acid	0.64 ± 0.01	nd	0.92 ± 0.01	nd	nd	0.47 ± 0.02	0.13 ± 0.04
Total	9.40 ± 0.09 b	6.09 ± 0.04 f	7.60 ± 0.05 d	5.10 ± 0.12 g	10.07 ± 0.03 a	9.06 ± 0.09 c	7.26 ± 0.08 e

Values are given as mean (*n* = 2) ± standard deviation (absolute value). Same letters indicate similar values (*p* < 0.05). nd = Not detected.

**Figure 2:**
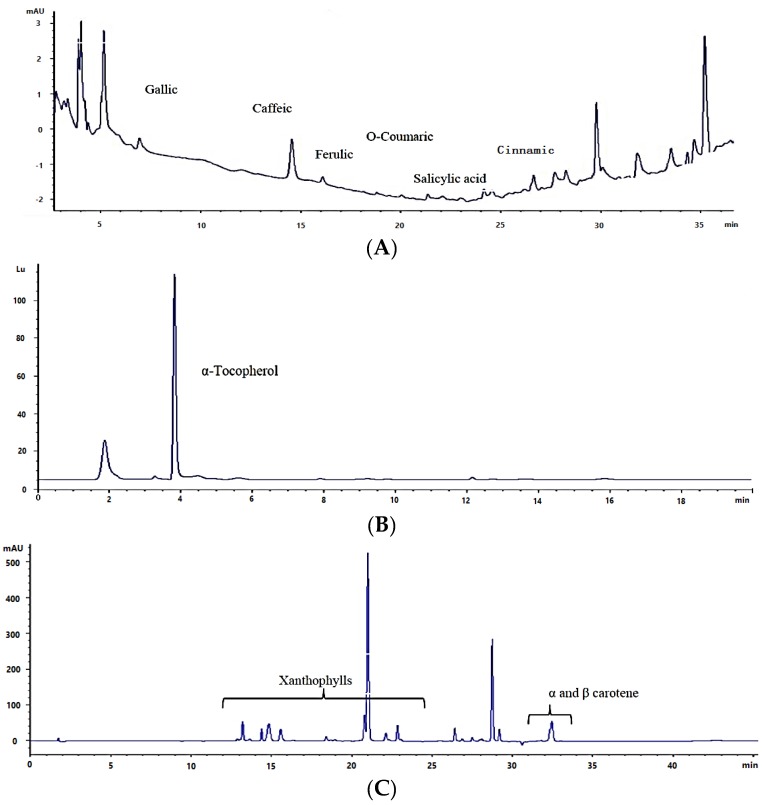
HPLC chromatograms of (**A**) phenolic compounds; and (**B**) tocopherols and (**C**) carotenoids.

As shown in Figure 2A there are other unidentified compounds, which might stem from flavonoids with complex structure. It has been shown that coumaric acid, which is the precursor of the flavonoid synthesis, is present in microalgae species. It has also been shown that the metabolic capacity for production of flavonoids is present in all major evolutionary lineages of microalgae and cyanobacteria [22]. Concentration and composition of the phenolic compounds in microalgae biomass could be affected by both species and growth conditions. Spectrophotometric assay confirmed the presence of flavonoids in the microalgae species grown on industrial wastewater (Table 1). Evaluation of the relationship between total phenolic compounds and total flavonoids indicates that the correlation based on *R*^2^ just explains 11.7% of the variability in total phenolic compounds, demonstrating a weak relationship between total phenolic and flavonoids. The reason might be that the Folin reagent does not only measure phenols, and can generally react with other reducing substances. It, therefore, measures the total reducing capacity of a sample, including some nitrogen-containing compounds, metal complexes, vitamin derivatives, and organic acids [36]. While spectrophotometric analysis of flavonoids is a more specific method. Furthermore in a complex sample, the interference from other compounds, e.g., pigments can affect the precision of the result of spectrophotometric analysis of flavonoids.

### 2.3. Total Tocopherols

Total tocopherol content in methanolic extracts varied highly between the species (Table 1), with highest amount in De.S (361.9 ± 23.1 µg/g) and lowest in P.T (13.12 ± 0.01 µg/g). There was no statistically significant difference between C.S1 and C.S2 so the light intensity did not significantly affect the amounts of tocopherols in these samples. Tocopherol composition mostly includes α-tocopherol (Figure 2B) in all samples. As shown in Table 1, total tocopherol content was higher in some green algae (Chlorophyceae) compared to others (Eustigmatophyceae and diatoms). Only few publications reported the tocopherol composition of microalgae. The effects of nitrogen source, concentration, and growth phase on tocopherol concentration in *Nannochloropsis occulata* was reported by Durmaz *et al.* [37]. In this research high amount of α tocopherol was reported as 2326 ± 39 μg/g DW for this specie. Other studies reported total tocopherols as 283.6 μg/g, 153.2 μg/g, and 157.7 μg/g for *E. gracilis, Dunaliella salina*, and *Tetraselmis suecica,* respectively [38], and 421.8 μg/g, 58.2 μg/g, 116.3 μg/g, and 669.0 μg/g of α-tocopherol for *Tetraselmis suecica, Isochrysis galbana, Dunaniella tertiolecta*, and *Chlorella stigmatophora*, respectively [39]. Our results for De.S are comparable to these reports showing the potential of the this specie for production of tocopherol when cultivated on industrial wastewater.

### 2.4. Total Carotenoids and Carotenoid Composition

Total carotenoids varied highly between species (Table 1). Highest amounts of total carotenoids were detected for De.S and lowest for N.L, at 6.70 ± 0.017 mg/g, and 2.56 ± 0.02 mg/g, respectively. Light intensity had statistically significant effect on the amounts of carotenoids in *Chlorella sorokiniana.* Thus, when exposed to high light intensity carotenoid production increased to 4.98 ± 0.07 mg/g in C.S1, compared to C.S2 with 2.92 ± 0.15 mg/g. Carotenoids accumulation can be affected by growth conditions e.g., light intensity and growth media composition, so again it can be considered that compatibility of *Desmodesmus* sp. to the growth conditions resulted in higher accumulation of carotenoids. Total carotenoid content in the biomass falls within the range given previously by several reports [15,16,40,41,42]. It has been shown that both carotenoid content and composition can be influenced by culture conditions such as growth media composition [16]. We used industrial wastewater as a source of nutrients, so comparison of the results with other studies shall be done with this consideration.

Carotenoid compositions of the strains also varied between different classes (Table 3).The main carotenoid compound in samples from green algae class was lutein with the amounts 5111 µg/g, 3014 µg/g, 3220 µg/g, and 2069 µg/g for De.S, Du.S, C.S1, and C.S2, respectively. In both samples N.S and N.L, which belong to Eustigmatophyceae class, the main carotenoid was violaxanthin at 1679 µg/g in N.S and 1228 µg/g in N.L. In diatom P.T the most abundant carotenoids were diadinoxanthin and diatoxanthin as 2166 µg/g and 1558 µg/g, respectively. The highest accumulation of β-carotene was detected in N.S, followed by C.S1 and Du.S as 2223 µg/g, 1039 µg/g, and 743 µg/g, respectively. *Dunaniella salina* is famous for its ability to accumulate β-carotene [11], but our results do not confirm that. The fact that production of β-carotene can be highly affected by suboptimal growth conditions such as growth media composition, starvation, and light intensity was shown in different studies [28] and could explain why Du.S did not accumulate high levels of β-carotene. Astaxanthin, antheraxanthin, 19-butyro-fucoxanthin, and cantaxanthin were detected just in C.S1, Du.S, P.T, and N.S, respectively. It has been shown previously that lutein and β-carotene prevailed among carotenoids in *Desmodesmus* microalgae followed by xanthophylls of the violaxanthin cycle (violaxanthin, antheraxan, zeaxanthin) and neoxanthin that are typical of Chlorophyta family [34].

**Table 3:** Pigments composition of microalgae biomass. Amounts are represented in (µg/g) unidentified peaks are less than 5% of total pigments for all samples.

Pigments (µg/g)	De.S	Du.S	N.L	P.T	N.S	C.S1	C.S2
Chlorophyll c3	nd	nd	nd	nd	nd	nd	nd
Unknown	212.2 ± 5	351.0 ± 7.5	nd	nd	nd	269.7 ± 4.6	80.78 ± 1.3
Chlorophyllide	nd	nd	nd	nd	nd	nd	nd
Peridin	nd	nd	nd	nd	nd	16.70 ± 0.1	4.69 ± 0.1
Vaucheriaxanthin	nd	nd	164.8 ± 2.6	nd	85.16 ± 0.6	nd	nd
19-But-Fucoxanthin	nd	nd	nd	50.75 ± 2.2	nd	nd	nd
Fucoxanthin	nd	nd	183.2 ± 9.8	264.5 ± 29	13.05 ± 0.1	104.8 ± 5.5	22.2 ± 0.2
Neoxanthin	158.3 ± 2.5	103.2 ± 12	423.4 ± 28	nd	53.45 ± 3.2	48.29 ± 1.2	20.0 ± 0.1
Prasinoxanthin	nd	nd	nd	nd	nd	22.64 ± 0.20	41.13 ± 0.2
Violaxanthin	54.60 ± 2.3	83.01 ± 11.7	1228 ± 61	nd	1679 ± 83	nd	nd
19-hex-fuco	nd	nd	nd	nd	nd	nd	nd
Dinoxanthin	nd	nd	nd	nd	nd	nd	nd
Antheraxanthin	nd	344.4 ± 4.6	nd	nd	nd	nd	nd
Astaxanthin	nd	nd	nd	nd	nd	48.42 ± 0.5	nd
Diadinoxanthin	256.7 ± 3	43.44 ± 1.0	nd	2166 ± 68	140.5 ± 2.4	nd	nd
Alloxanthin	17.64 ± 0.1	55.73 ± 0.5	nd	nd	130.5 ± 2.9	94.06 ± 0.8	24.33 ± 0.1
Diatoxanthin	26.75 ± 1.0	nd	136.3 ± 1.0	1558 ± 88	nd	nd	nd
Lutein	5111 ± 61	3014± 76	nd	nd	nd	3220 ± 54	2069 ± 34
Zeaxanthin	284.5 ± 2.5	195.8 ± 6.7	136.8 ± 1.0	nd	584.9 ± 3.6	151.1 ± 1.7	15.52 ± 1.0
Dihydro lutein	145.0 ± 2.9	175.6 ± 2.5	nd	216.2 ± 2.0	nd	248.7 ± 9	111.1 ± 17
Unknown	nd	nd	nd	nd	165.2 ± 3.5	nd	nd
Chlorophyll b	862.6 ± 8.6	1454 ± 23	nd	nd	nd	725 ± 35	389.3 ± 19
Chlorophyll a	2993 ± 14	3424 ± 87	1065 ± 22	2714 ± 23	2001 ± 54	615.3 ± 4.1	1455 ± 3.9
β-carotene	647.3 ± 13	743.5 ± 44.3	284.5 ± 2	348.7 ± 4.4	2223 ± 88	1039 ± 17	614.4 ± 8.3
Canthaxanthin	nd	nd	3.40 ± 0.05	nd	136.5 ± 12.1	nd	nd
α-carotene	nd	76.01 ± 0.1	nd	nd	84.16 ± 3.2	nd	nd

Values are given as mean (*n* = 2) ± standard deviation (absolute value). nd = Not detected.

### 2.5. Antioxidative Properties

#### 2.5.1. ABTS-Radical Scavenging Capacity (TEAC)

This method monitors the ability of antioxidant compounds to interfere with the reaction between peroxy radicals. This assay involves the initiation of peroxidation by generating water-soluble peroxyl radicals and is sensitive to all known chain breaking antioxidants such as phenolics and carotenoids [43].

Highest TEAC radical scavenging activity in this study was detected in De.S and the lowest in P.T. There was no statistically significant difference between concentrations 0.25 mg/mL and 0.5 mg/mL. (Table 4). Results of radical scavenging for C.S1, which was exposed to higher light intensity was higher than for C.S2, which can be explained by the higher content of carotenoids in this sample. Based on the results of analysis of variance, phenolic compounds showed highest contribution to the ABTS radical scavenging capacity, followed by carotenoid contents. We estimated the correlation of independent variables including tocopherols, phenolic compounds, and carotenoids to the results of this assay. The following fitted model was calculated and can explain *R*^2^ = 86.06% of the variability in assay’s results.

TEAC = −1.28 + 1.13 (Phenolic Compounds) + 0.343 (Carotenoids) + 0.0017 (Tocopherols)

**Figure 3:**
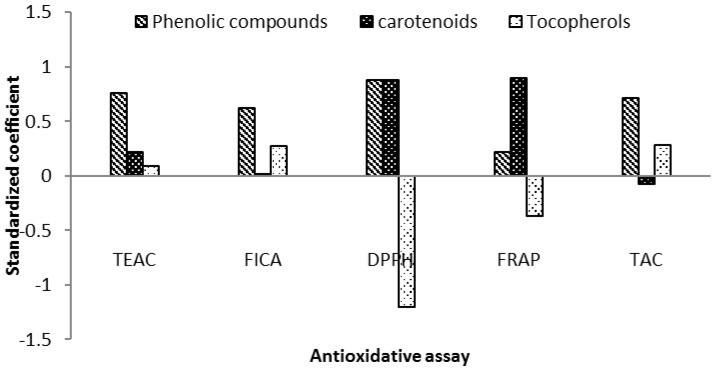
PLS coefficient plots. Bars represent the standardized correlation coefficients of predictor variables (phenolic compounds, tocopherols, and carotenoids) for each response variable (antioxidative assay).

Results of the multivariate data analysis, which was done by the Partial Least Squares (PLS) method, also confirmed that phenolic compounds and carotenoids were major contributors to the ABTS radical scavenging capacity in microalgae methanol extracts (Figure 3). Carotenoids, such as lutein and zeaxanthin, are known for their ability to quench the radicals and singlet oxygen [44], so this can explain the contribution of carotenoids in this radical scavenging assay. This assay have been used previously by many different studies to evaluate the microalgae radical scavenging capacity [3,16] and our results are in agreement with the results of these reports.

#### 2.5.2. Ferrous Ion-Chelating Ability (FICA)

The FICA is a measure of chelating ability of the ferrous ion, which is important to avoid reactions that could lead to development of radicals such as hydroxyl. The following multiple regression model was calculated to show the correlation of phenolics, carotenoids, and tocopherols to the results of ferrous ion chelating ability.

FICA = 3.26 + 1.42 (Phenolic Compounds) + 0.046 (Carotenoids) + 0.008 (Tocopherols)

The R-squared statistics indicates that the fitted model explains 68.56% of the variability in chelating ability. Analysis of variance confirmed that phenolic compounds is the main contributor to the results (*F* = 2.74, *p* = 0.02). Multivariate data analysis based on PLS coefficient method also confirmed the order of contribution as phenolic compounds and tocopherols, respectively (Figure 3). Highest chelating ability was detected in De.S (1 mg/mL) and lowest activity in P.T (0.25 mg/mL). Chelating ability in sample of *Chlorella sorokiniana,* which was exposed to higher light intensity (C.S1), was lower, compared to C.S2 (Table 4). Our result based on this assay confirmed some previous studies [9,13].

**Table 4:** Result of evaluation of antioxidative properties.TEAC (Trolox equivalent/g). FICA (% chelating). DPPH (% inhibition). FRAP (mg/g Ascorbic acid equivalent) and TAC(GAE/g).

Concentration * ^TEAC^	**De.S a**	**Du.S c,d**	**N.L c,d**	**P.T e**	**N.S a,b**	**C.S1 d**	**C.S2 b,c**
1.0 a	24.26 ± 0.60	14.38 ± 0.40	16.60 ± 0.20	6.79 ± 0.02	20.34 ± 0.15	18.75 ± 0.16	13.48 ± 0.38
0.50 b	10.05 ± 0.30	6.58 ± 0.03	5.48 ± 0.23	3.35 ± 0.10	8.68 ± 0.10	6.32 ± 0.11	7.30 ± 0.20
0.25 b	7.05 ± 0.10	5.01 ± 0.05	5.16 ± 0.01	2.73 ± 0.20	6.66 ± 0.14	5.24 ± 0.14	6.80 ± 0.010
Concentration ^FICA^	De. S a	Du.S c	N.L d	P.T e	N.S b	C.S1 c,d	C.S2 b
1.0 a	20.10 ± 0.51	13.99 ± 0.18	11.61 ± 0.97	9.67 ± 0.60	16.90 ± 0.14	12.15 ± 0.042	17.91 ± 0.21
0.5 b	16.87 ± 0.60	11.77 ± 0.31	9.45 ± 0.49	7.55 ± 0.21	14.25 ± 0.35	10.57 ± 0.60	14.92 ± 0.17
0.25 c	8.57 ± 0.17	5.42 ± 0.17	3.95 ± 0.21	3.35 ± 0.21	7.17 ± 0.10	5.40 ± 0.14	7.53 ± 0.19
Concentration ^DPPH^	De. S c	Du.S d	N.L a,b	P.T d	N.S b	C.S1 a	C.S2 c,d
1.0 a	29.11 ± 0.01	26.95 ± 0.10	35.17 ± 0.08	28.35 ± 0.07	30.32 ± 0.02	34.09 ± 0.08	28.06 ± 0.04
0.5 b	18.24 ± 0.14	15.55 ± 0.21	19.44 ± 0.14	15.58 ± 0.01	21.26 ± 0.08	26.05 ± 0.07	15.38 ± 0.26
0.25 c	10.29 ± 0.24	12.66 ± 0.09	14.27 ± 0.09	8.41 ± 0.12	12.55 ± 0.07	11.74 ± 0.05	9.34 ± 0.15
Concentration ^FRAP^	De.S a	Du.S b,c	N.L e	P.T d	N.S a	C.S1 c,d	C.S2 b
1.0 a	0.45 ± 0.01	0.31 ± 0.02	0.30 ± 0.01	0.27 ± 0.01	0.39 ± 0.021	0.32 ± 0.01	0.35 ± 0.01
0.5 b	0.32 ± 0.01	0.27 ± 0.01	0.16 ± 0.01	0.26 ± 0.01	0.35 ± 0.02	0.25 ± 0.01	0.27 ± 0.01
0.25 c	0.25 ± 0.01	0.25 ± 0.01	0.14 ± 0.01	0.17 ± 0.01	0.30 ± 0.01	0.18 ± 0.01	0.22 ± 0.01
Time ** ^TAC^	De.S a	Du.S c	N.L b	P.T d	N.S a	C.S1 c	C.S2 c
60 a	5.22 ± 0.14	3.24 ± 0.13	2.65 ± 0.13	1.83 ± 0.01	6.30 ± 0.012	2.96 ± 0.08	3.47 ± 0.04
90 b	8.56 ± 0.21	5.17 ± 0.05	6.57 ± 0.41	2.55 ± 0.21	7.29 ± 0.13	4.32 ± 0.09	4.65 ± 0.16
**120 b**	**8.95 ± 0.01**	**5.27 ± 0.01**	**7.00 ± 0.02**	**2.99 ± 0.06**	**7.30 ± 0.14**	**4.31 ± 0.26**	**5.36 ± 0.06**

Values are given as mean (*n* = 3) ± standard deviation (absolute value). For each test the same letters indicate homogeneous values (*p* < 0.05). * Values in the first column show the concentration of methanolic extract for TEAC, FICA, DPPH, and FRAP (mg algae biomass dry weight/mL). ** Values in the first column show the reaction time (min) for TAC.

#### 2.5.3. DPPH Radical Scavenging Activity Assay (DPPH)

This assay is based on the measurement of the reducing ability of antioxidants toward DPPH. The ability can be evaluated by electron spin resonance or by measuring the decrease of its absorbance. The 1,1-diphenyl-2-picryl-hydrazil (DPPH) radical scavenging activities of methanolic extracts increased with increasing concentration. Result of analysis of variance showed a statistically significant effect of both concentration and species on DPPH radical scavenging activity at the 95.0% confidence levels (Table 4). The highest activity was detected for N.L and C.S1 at 1 mg/mL and the lowest activity for P.T at concentration of 0.25 mg/mL (Table 4). There was a significant difference between the C.S1 and C.S2 samples, showing that the light intensity had a considerable effect on the radical scavenging property. A multiple linear regression model was fitted (*R*-squared = 69.86%) to describe the relationship between DPPH and independent variables including tocopherols, carotenoids, and phenolics as below.

DPPH = −1.62 + 2.39 (Phenolic Compounds) + 2.36 (Carotenoids) − 0.04 (Tocopherols)

Based on results of analysis of variance for the fitted model, both carotenoids and phenolic compounds showed strong correlation to the DPPH radical scavenging property of microalgae methanolic extracts. This finding was confirmed by using PLS correlation method, as shown in Figure 3. Contribution of the carotenoids to the radical scavenging activity was described by several researches previously [12,45,46]. Our results confirm that both carotenoids and phenolics are contributing to the radical scavenging property of microalgal methanolic extract, while the contribution of tocopherols was not estimated as significant.

#### 2.5.4. Ferrous Reduction Power (FRAP)

The highest reducing power was detected in De.S (1 mg/mL) methanolic extract and the lowest in N.L (0.25 mg/mL). Both effects of concentration and species were evaluated as significant at 95% confidence level. Multiple comparison procedure based on Fisher’s least significant difference (LSD) procedure revealed that there was a statistical significant difference between concentrations, and strains (Table 4). The following regression model was calculated to describes the relationship between results of FRAP assay and three independent variables including carotenoids, phenolic compounds, and tocopherols.

FRAP = 0.059 + 0.0095 (Phenolic Compounds) + 0.038 (Carotenoids) − 0.00019 (Tocopherols)

There was a statistically significant relationship between the variables at the 95.0% confidence level. The *R*-Squared statistic explains 55.64% of the variability in the results of FRAP assay. Highest effect corresponded to carotenoids (*T* = 2.9 and *p*-value = 0.01) followed by phenolic compounds which can suggest the carotenoid as the main contributor to the ferrous reducing power. It was also confirmed by PLS test as shown in Figure 3. The effect of light intensity on the two *Chlorella* species was also evaluated as significant. The correlation between content of carotenoids to the ferrous reduction power was already reported before [13]. The antioxidant mechanism of carotenoids is mostly known as radical scavenging, so it can show the individual ability of carotenoids, such as lutein, to involve in single electron transfer-based reactions which are the basic principle in the analysis of ferric reducing power assay [44].

#### 2.5.5. Total Antioxidant Capacity Assay (TAC)

This method can determine the antioxidant capacity, through the formation of phosphomolybdenum complex, and the reduction of Mo (VI) to Mo (V) by the antioxidant components in sample which would result in formation of a green phosphate Mo (V) complex at acidic pH. Reaction time is normally between 60 to 150 min, depending of the composition in the extracts as the formation of the complex is temperature-dependent for various reducing compounds, such as tocopherols and phenolic compounds. The analysis of variance with two factors (species and time of reaction) was used to evaluate the variability of total antioxidative capacity. Result showed a statistically significant effect of both variables on total antioxidative capacity at the 95.0% confidence levels. A multiple comparison procedure based on Fisher’s LSD procedure revealed that there is a statistically significant difference between strains, while the difference for the reaction time was not significant between 90 and 120 minuutes (Table 4). Thus, the reaction can be completed in 90 min. Highest activity was detected for De.S at 120 min and lowest activity for T at 60 min with values of 8.95 ± 0.07 mg GAE/g and 1.83 ± 0.01 mg GAE/g, respectively. There was no significant difference between the C.S1 and C.S2 samples (Table 4).

A multiple linear regression model was calculated which describes the relationship between total antioxidative capacity and the three independent variables including carotenoids, phenolic compounds, and tocopherols. The equation of the fitted model was:

Total Antioxidative Capacity = −0.011 + 1.0 (Phenolic Compounds) − 0.10 (Carotenoids) + 0.005 (Tocopherols)

The *R*-Squared statistic indicates that the model as fitted explains 77.49% of the variability in total antioxidant capacity. Further analysis of variances showed that phenolic compounds have the main effect in results of total antioxidative capacity. It could be attributed to the fact that both methods (TAC and Folin) are based on the reaction of reducing compounds in the extract. We used gallic acid as a reference standard for both tests and this can be another reason for this finding. Contribution of tocopherols and carotenoids in the result of this assay were estimated as weak and not significant. Further confirmation of the results was done by estimation of correlations by PLS coefficient at 95% confidence level. As shown in Figure 3, phenolic compounds were the main correlating factor for total antioxidative capacity assay in microalgae samples. The same result was reported previously [34] while another study reported the contribution of both carotenoids and phenolic compounds [7].

### 2.6. Contribution of Carotenoids in Antioxidative Activity of Microalgae Biomass

Our results showed that carotenoids contribute significantly to some antioxidant properties of microalgae species grown on industrial waste water. The contribution of carotenoids in antioxidant activity of microalgae extracts was evaluated in some previous studies [9,34,47].

The antioxidative power of carotenoids is not the same. The electron-rich conjugated system of the polyene and functional cyclic end groups determine the antioxidant activities of carotenoids together [47]. Ketocarotenoids, including astaxanthin and canthaxanthin, can be found mostly in algae. Epoxy carotenoids such as antheraxanthin, violaxanthin and fucoxanthin are also abundant in algae. It has been shown that pigments like astaxanthin, β-carotene, lutein, neoxanthin, and also zeaxanthin have a scavenging property [47], while astaxanthin has been claimed to show the highest effect among all carotenoids. Scavenging function of carotenoids against peroxyl radicals (ROO*) was reported as even stronger than α-tocopherol with order of astaxanthin > lutein > zeaxanthin > α-tochopherol > fucoxanthin > β-carotene while in relation to the hydroxyl radical (HO) scavenging capacity the order of strength was reported as β-carotene > lutein > zeaxanthin > astaxanthin > α-tochopherol [47]. It was claimed that the *in vitro* antioxidant property of astaxanthin was 10 times stronger than zeaxanthin, lutein, tunaxanthin, canthaxanthin, and β-carotene, and even 100 times stronger than α-tocopherol [48]. In our study both composition and content of carotenoids varied significantly between the species. Main carotenoid compound(s) in green algae including De.S, Du.S, C.S1, and C.S2 was lutein, while in P.T, and *Nannochloropsis* samples the main carotenoids were diatoxanthin and diadinoxanthin, and violaxanthin and β-carotene, respectively.

### 2.7. Effects of Source of Nitrogen on Productivity of Biomass and Bioactive Compounds

Nitrogen is the most important nutrient for the growth of the algal biomass, and is a key constituent of many algal cellular components. Most microalgae species are capable of utilizing a variety of inorganic nitrogen (e.g., ammonia, nitrate, and nitrite, *etc.*) [46], so waste water from different sources such as industrial and municipal activities could be used as a good nitrogen sources. In the industrial wastewater we used in this study the majority of nitrogen is in the form of ammonia. Among the above nitrogen forms, ammonium is the most preferred form of nitrogen source for some microalgae in part because its uptake and utilization by microalgae is most energy efficient [11,21]. It is commonly accepted that nitrogen metabolism is linked to carbon metabolism in algae because they share organic carbon and energy supplied directly from photosynthetic electron transport and CO2 fixation as well as from the metabolic pathway of organic carbon [23,46]. Biomass is the main product of a microalgae cultivation system [24], and productivity of intracellular bioactive compounds, such as carotenoids and phenolics depends directly on the productivity of the biomass. The literature shows that the cell growth rate, lutein content, and lutein productivity of some microalgae are mainly influenced by the culture conditions, such as light intensity and nitrogen concentration [6,34,42]. Green algae have a fast growth rate with a good compatibility to the growth conditions and for this reason they have a wide potential for large scale cultivation, because of their robustness and simple nutritional requirements, so the higher concentration of carotenoids (lutein) in fresh water *Desmodesmus* sp. could be correlated to this feature. In addition to light, which is the main energy source of microalgae, chemicals including carbon dioxide, inorganic nitrogen (ammonia or nitrate), and phosphate are required for photoautotrophic growth. Production of lutein in *Chlorella sorokiniana* or *Desmodesmus* sp. at higher concentrations has been demonstrated before [41,42]. For the higher production of lutein, in addition to the overexpression of specific enzymes, additional storage space (outside of the photosystem) needs to be created. Therefore, *Desmodesmus* sp. and *Chlorella sorokiniana* are suitable strains for lutein production by using industrial wastewater as main nutrient source.

## 3. Experimental Section

### 3.1. Chemicals and Reagents

Standards of phenolic compounds and tocopherols were purchased from Sigma (St. Louise, MO, USA) and Fluka (Deisenhofen, Germany) and standards of pigments were purchased from DHI (Hørsholm, Denmark). HPLC grade acetonitrile, heptane, isopropanol, methanol, and acetone were purchased from Sigma and Fluka. HPLC grade water was prepared at DTU Food using Milli-Q^®^ Advantage A10 water deionizing system from Millipore Corporation (Billerica, MA, USA).

### 3.2. Microalgae Biomass

Microalgae strains *Nannochloropsis salina.* (*Strain Number: 40.85*) and *Nannochloropsis limnetica* (*Strain Number: 18.99*) were obtained from the culture collection of algae (SAG), University of Gottingen. Strain of *Desmodesmus* sp. *(*De.S*)* was isolated from waste water treatment system, Kalundborg Kommune and identified by Dr Gert Hansen, Department of Biology, University of Copenhagen. *Chlorella sorokiniana (*C.S1 and C.S2*)* were cultivated in flat panel reactors at two light intensities, 2000 µmol photon m^−2^·s^−1^ and 200 µmol photon m^−2^·s^−1^, respectively, as in [49]*, Phaeodactylum tricornutom (*P.T*)* and *Dunaliella salina (*Du.S*)* were cultivated at DTU Environment in 10 L Schott bottles, stirred with magnets and aerated with 2% carbon dioxide/air mixture under fluorescent lights with intensity 300 µ mol photon m^−2^·s^−1^. *Nannochloropsis salina (*N.S), *Desmodesmus* sp. and *Nanochloropsis limnetica (*N.L) were cultivated at DTU Food in 5 L Schott bottles, stirred with magnets and aerated with 2% carbon dioxide/air mixture under fluorescent lights with intensity 200 µmol photon m^−2^·s^−1^. Industrial waste water was obtained from Kalundborg municipality and used as main nutrient source for all cultivations. Table 5 shows specification of industrial wastewater. Microalgae biomass was harvested at stationary phase by a pilot scale (100 L/h) ceramic membrane microfiltration unit with one micron pore size membrane (Liqtech International, Ballerup, Denmark) and then immediately freeze dried. Dried samples were stored at −20 °C until analysis.

**Table 5:** Chemical composition of industrial wastewater which was used as main nutrient source.

Item	Unit	Amount
pH	-	8.1
Suspended solids	mg/L	20
Total N	mg/L	190
Ammonia + ammonium-N	mg/L	150
Nitrite + nitrate	mg/L	<0.1
Total P	mg/L	11
Sulphate	mg/L	3.6
Total cyanide	µg/L	2.5
Total Alkalinity	mmol/L	62.5
EDTA	mg/L	<0.5
Sodium(Na)	mg/L	1500
Cadmium (Cd)	µg/L	<0.05
Copper (Cu)	µg/L	3.4
Iron (Fe)	mg/l	0.23
Cobolt (Co)	µg/L	<0.5

### 3.3. Sample Preparations

#### 3.3.1. Antioxidative Properties, Tocopherols, and Phenolic Compounds (HPLC)

Freeze-dried samples were ground into a fine powder, and then 50 mg samples were soaked in 5 mL of pure methanol and shaken vigorously for 30 s. Then tubes were put in sonication bath (Branson Corp., Danbury, CT, USA) in the dark and at room temperature for 45 min. Then, all samples were centrifuged at 7500 *g* for 10 min and supernatants were separated. The extraction process was repeated with another 5 mL portion of pure methanol. Collected supernatants were combined and stored at −20 °C. For analysis of tocopherols, one milliliter of the extract was evaporated under a stream of nitrogen and then re-dissolved in one milliliter of *n*-heptane. For analysis of phenolics and antioxidative properties tests, the rest of the methanolic extract solution was diluted with pure methanol to various concentrations (mg algae biomass dry weight/mL) for each test.

#### 3.3.2. Pigments

Freeze-dried samples were ground into a fine powder, after which 20 mg samples were soaked in 5 mL of methanol containing 0.025 µg/mL BHT as internal standard and antioxidant. Then, the tubes were shaken vigorously for 30 seconds and put in sonication bath at a temperature lower than 5 °C for 15 min. Subsequently, the tubes were centrifuged at 5000 *g* and the supernatants were separated. The extraction was repeated with 5 mL portion(s) of solvent until a nearly colorless biomass was obtained. Supernatants were combined and used immediately for the analysis.

### 3.4. Analytical Methods

#### 3.4.1. Total Phenolic Content

The total phenolic content of the algae extracts was determined via a modified Folin–Ciocalteu method as described by Choochote *et al.* [9]. Briefly, 100 μL of diluted extract solution (1 mg/mL) was mixed with 0.6 mL of deionized water and 0.5 mL of Folin-Ciocalteu reagent in a test tube and then 1.5 mL of 20% sodium carbonate aqueous solution was added and the volume was made up to 10 mL with deionized water. The samples were incubated for 30 min at room temperature in darkness and then absorbance of the reaction mixtures were measured at 760 nm. Gallic acid was used as a standard and the total phenolic content of the extracts were expressed in milligram gallic acid equivalent.

#### 3.4.2. Phenolic Compounds (HPLC)

Extracts (2.3.1) were filtered prior to the analysis by 0.22 µm PVDF syringe filter, and then analyzed by HPLC using an Agilent 1100 Liquid Chromatograph (Agilent technologies, Santa Clara, CA, United States) equipped with a DAD. The separation was carried out on a Prodigy ODS-3 column 250 mm, 46 mm with 5 µm particle size from Phenomenex (Torrance, CA, USA). Injection volume was 20 µL and the mobile phase was a mixture of solvent A (phosphoric acid in de-ionized water, pH = 3) and solvent B (acetonitrile) at 0.9 mL/min. The gradient started with 5% of B and after 2 min increased to 40% in 20 min and again increased to 100% B at 15 min and finally kept constant for 25 min. Total acquisition time was 70 min. Detection was done at 280 nm. The identification of the peaks was done using standards which include gallic acid, 2,5-dihydroxy benzoic acid, 3,4-dihydroxy benzoic acid, chlorogenic acid, catechin hydrate, ginnestein, 4-hydroxy benzoic acid, caffeic acid, syrringic acid, *p*-coumaric acid, ferulic acid, *O*-salicylic acid, and cinnamic acid. Total of identified phenolics was also calculated.

#### 3.4.3. Total Carotenoids and Pigment Composition

The extracts (2.3.2) were filtered prior to the analysis by methanol compatible 0.22µm PTFE syringe filter and then analyzed by HPLC using an Agilent 1100 Liquid Chromatograph equipped with a DAD. The separation was carried out in a Zorbax Eclipse C8 column 150 mm, 46 mm with 3.5 µm particle size from Agilent. The chromatographic separation was carried out according to the method described by Van Heukelem *et al.* [50] with modifications. The temperature of injection port was 5 °C.

The mobile phase was a mixture of solvent A (70% methanol + 30% of 0.028 M tertiary butyl ammonium acetate in water) and solvent B (methanol) at a flow rate of 1.1 mL/min. The gradient program was started with 5% of B and then increased to 95% in 27 min, kept constant for 7 min and then changed to 100% in one minute and kept constant for 5 min. Total acquisition time was 40 min. The temperature in the injection port was kept constant at 5 °C and the sample was mixed with the buffer (0.028 M tertiary butyl ammonium acetate in water) at the proportion of 1:3 for 3 min just prior to the injection. Identification of peaks and calibration was done by individual standards for each pigment. Detection was done at 440 nm for pigments and 280 nm for BHT as internal standard. Sum of the carotenoids was calculated as total carotenoids.

#### 3.4.4. Total Flavonoids

Total flavonoids content in algae extracts was determined by the method described by Sava *et al.* [29], with some modifications. To 20 μL of algal extract, 20 μL 10% AlCl_3_ and 20μL 1 M potassium acetate plus 180 μL of distilled water was added, and then tubes were kept at room temperature for 30 min. Optical density was measured at 415 nm against blank. The calibration curve was made by quercetin prepared in methanol. Results expressed as milligrams of quercetin equivalent per gram of sample.

#### 3.4.5. Total Tocopherols

One milliliter of the methanolic extract was evaporated to dryness in darkness and under a stream of nitrogen and then re-dissolved in a mixture of isopropanol: heptane (0.5:99.5, *v*/*v*). Then the solution was filtered by suitable 0.22 µm PTFE syringe filter and 20 µL of filtrate was injected to HPLC. Analysis was done based on the AOCS official method as [51] using an Agilent 1100 Liquid Chromatograph equipped with a fluorescence detector, with the excitation wavelength set at 290 nm and emission wavelength at 330. The separation was carried out in isocratic mode by Spherisorb column 150 mm, 46 mm with 3 µm particle size, using a mixture of isopropanol: n- heptane (0.5:99.5, *v*/*v*) as mobile phase.

#### 3.4.6. Total Antioxidant Capacity Assay (TAC)

The total antioxidant capacity assay of the microalgae extracts was determined by the method of Pan *et al.* [52] with some modifications. 300 μL of diluted extract solution (0.5 mg/mL) were added to a test tube containing 3 mL reagent solution (0.6 M sulfuric acid, 28 mM sodium phosphate plus 4 mM ammonium molybdate). The reaction mixture was incubated at 95 °C for 60, 90 or 120 min. Then the mixtures were cooled to room temperature and absorbance was measured at 695 nm against water as blank. Gallic acid was used as the reference standard.

#### 3.4.7. DPPH Radical Scavenging Activity Assay (DPPH)

The method used for measuring the DPPH radical scavenging ability of the algae extracts was that of Choochote *et al.* [9]. Various concentrations (0.25, 0.5, and 1 mg/mL) of the extract was made with pure methanol and then 100 µL of each extract was added to 2 mL of DPPH (0.5 mM in absolute ethanol), respectively. The mixtures were shaken vigorously and then left to stand at room temperature for 30 min in the dark. The absorbance was measured at 517 nm against a methanol extract/water extract blank. BHT was used as the reference standard and results compared as the percent of inhibition against 100% of inhibition. The percent inhibition (I %) was calculated using the formula:

I % = ((Abs control − Abs sample)/Abs control) × 100

#### 3.4.8. Ferrous Ion-Chelating Ability (FICA)

The ferrous ion-chelating ability was determined according to the method of Duan *et al.* [31]. The extract solution was diluted with pure methanol to various concentrations (0.25, 0.5, and 1 mg/mL) and then 2000 μL of each was mixed with 2.7 mL distilled water, FeCl2 (0.1 mL, 2 mM) and ferrozine (0.2 mL, 5 mM). Then, the solutions were incubated for 10 min in dark and at room temperature. After incubation, the absorbance was measured at 562 nm. Deionized water (2 mL) was used instead of sample as a control, and instead of ferrozine solution as a blank. EDTA (1 mg/mL) was used as reference. The ferrous ion-chelating ability was calculated as follows:

Chelating ability (%) = [*A*_0_ − (*A*_1_ − *A*_2_)/*A*_0_] × 100, where *A*_0_ is the absorbance of the control, *A*_1_ the absorbance of the sample or EDTA, and *A*_2_ is the absorbance of the blank.

#### 3.4.9. Ferrous Ion Reduction Power (FRAP)

Reducing power of algae extracts were determined by the method of Benzie *et al.*, [53]. In brief, 1.0 mL of diluted extracts (0.25, 0.5 and 1 mg/mL) were mixed with 2.5 mL of phosphate buffer (0.2 M, pH 6.6) and 2.5 ml of potassium ferric cyanide (1%). Reaction mixture was kept in a water bath at 50 °C for 20 min. After incubation, 2.5 mL of trichloroacetic acid (10% of TCA) was added and tubes were centrifuged at 2000 rpm for 10 min. From the upper layer, 2.5 mL solution was mixed with 2.5 mL distilled water and 0.5 mL of FeCl3 (0.1%). Absorbance of all the solution was measured at 700 nm. Ferric reducing antioxidant power is expressed as the reducing power compared to ascorbic acid (1 mg/g) as reference standard.

#### 3.4.10. ABTS-Radical Scavenging (TEAC)

The ABTS radical scavenging activity was determined according to the method described by Li *et al.* [3]. For the assay, ABTS^+^ radical cation was generated by preparing a solution of 7 mM ABTS and 2.45 mM potassium persulphate in deionised water. The reaction mixture was allowed to stand in the dark for 16 h at room temperature and was used in the same day. The ABTS^+^ solution was diluted with deionised water to give an absorbance of 0.700 ± 0.050 at 734 nm. The extract solution was diluted with pure methanol to various concentrations (0.25, 0.5 and 1 mg/mL) and then 100 μL of each diluted sample were mixed with 1.9 mL of diluted ABTS^+^ solution. After 10 min dark incubation at room temperature, the absorbance was measured at 734 nm. Trolox (0–25 µM) was used as a reference standard. Controls were included de-ionized water and ammonium acetate buffer instead of the reagents and pure de-ionized water and 96% ethanol instead of sample.

#### 3.4.11. Statistical Analyses

Measurements were carried out in triplicate unless otherwise stated and the results are given as mean values ± absolute standard deviations. Results were compared using ANOVA test with least squares’ post-test with significance level α = 0.05. Multiple regression and multivariate data analysis (partial least squares coefficient method) were also carried out to evaluate and demonstrate the effects of carotenoids, phenolic, and tocopherols as predictor variables on each individual antioxidative property test as response variable. Partial least squares coefficient graphs were used to show significance and the magnitude of the relationship between predictors and responses. All statistical analyses were done by STATGRAPHICS-centurion XVI software from Statpoint Technologies (Warrenton, MO, USA).

## 4. Conclusions

This study evaluated the antioxidative properties of microalgal methanolic extract by means of different assays and correlated the results to the content of some natural antioxidants which were present in the microalgae biomass. Phenolic compounds contributed to all the antioxidative properties measured while the contribution of carotenoids to these properties was confirmed for the 1,1-diphenyl-2-picryl-hydrazil (DPPH) radical scavenging activity assay, ferrous reduction power (FRAP) and ABTS-radical scavenging capacity. Tocopherols did not appear to contribute to the antioxidative activities to a significant extent.

The study focused on main natural antioxidants in the microalgae biomass while there are many other compounds which are known as antioxidants such as amino acids, polysaccharides, and quinoid compounds which could affect the antioxidative properties in microalgae biomass.

*Desmodesmus* sp. which was isolated from waste water treatment facility in Kalundborg, Denmark, produced the highest amounts of pigments, phenolics, and tocopherols and had the best antioxidative properties. *Phaeodactylum tricornutom* species showed the poorest antioxidative properties and had the lowest amounts of antioxidants and the highest accumulation of beta-carotene was observed in *Nannochloropsis salina.*

The effects of light intensity for one species (*Chlorella sorokiniana*) was also evaluated, showing that high light intensity could improve the development of carotenoids while it could have adverse or no effects on the antioxidative properties. The industrial processing waste water which was used in this study contained very low concentrations of heavy metals and hazardous materials (Table 5), and could represent a suitable and feasible source of nutrient for the production of bioactive compounds such as pigments and tocopherols.
